# Free energy dissipation enhances spatial accuracy and robustness of self-positioned Turing pattern in small biochemical systems

**DOI:** 10.1098/rsif.2023.0276

**Published:** 2023-07-05

**Authors:** Dongliang Zhang, Chenghao Zhang, Qi Ouyang, Yuhai Tu

**Affiliations:** ^1^ The State Key Laboratory for Artificial Microstructures and Mesoscopic Physics, School of Physics, Peking University, Beijing 100871, People’s Republic of China; ^2^ Center for Quantitative Biology and Peking-Tsinghua Center for Life Sciences, AAIC, Peking University, Beijing 100871, People’s Republic of China; ^3^ Department of Physics, University of Illinois, Urbana, IL 61801, USA; ^4^ IBM T. J. Watson Research Center, Yorktown Heights, NY 10598, USA

**Keywords:** Turing pattern, non-equilibrium thermodynamics, reaction–diffusion systems, energy-accuracy trade-off, robustness against cellular fluctuations

## Abstract

Accurate and robust spatial orders are ubiquitous in living systems. In 1952, Turing proposed a general mechanism for pattern formation exemplified by a reaction–diffusion model with two chemical species in a large system. However, in small biological systems such as a cell, the existence of multiple Turing patterns and strong noise can lower the spatial order. Recently, a modified reaction–diffusion model with an additional chemical species is shown to stabilize the Turing pattern. Here, we study non-equilibrium thermodynamics of this three-species reaction–diffusion model to understand the relationship between energy cost and the performance of self-positioning. By using computational and analytical approaches, we show that beyond the onset of pattern formation the positioning error decreases as energy dissipation increases. In a finite system, we find that a specific Turing pattern exists only within a finite range of total molecule number. Energy dissipation broadens this range, which enhances the robustness of Turing pattern against molecule number fluctuations in living cells. The generality of these results is verified in a realistic model of the Muk system underlying DNA segregation in *Escherichia coli*, and testable predictions are made for the dependence of the accuracy and robustness of the spatial pattern on the ATP/ADP ratio.

## 1. Introduction

Spatial regularity (order) and pattern formation are ubiquitous in living organisms. Examples can be found in all living organisms spanning a large range of spatial and temporal scales, which ranges from patterning in limb development [1], feathers and hair in the skins of birds and mammals [2], phyllotaxis in plants [3] to accurate positioning of the chromosomal origin of replication in bacteria [4]. These spatial patterns in living systems are the emergent properties of the underlying biochemical and transport processes. In 1952, Alan Turing [5] proposed a general mechanism for pattern formation in reaction–diffusion systems, which was explained by a simple model with two chemical species with different diffusion constants. As Turing showed, when diffusion constants and reaction rates satisfy certain conditions, the spatially homogeneous solution of the system becomes unstable and spatial patterns start to emerge. Experimental evidence for reaction–diffusion patterns following Turing mechanism have been found in various biological processes such as yeast cell polarity [6], glycolysis in yeast [7], EGFR-dependent chemotaxis [8], Min system underlying precise positioning of cell division [9], hair follicle organization [10] and MukBEF cluster formation in *Escherichia coli* [11], which we will study in detail later in this paper.

In Turing’s original work, noise was not considered. However, for biological systems, the size of the system and the number of molecules in the system are relatively small. As a result, dynamics underlying pattern formation in biological systems is subject to finite size effects and large stochastic fluctuations [12–16]. In particular, there is the ‘fine tuning problem’ for mode selection as multiple modes can be excited above onset in a small system [17,18]. Furthermore, there is the ‘robustness problem’ of maintaining the Turing pattern due to large noise in the underlying small biological systems [19,20]. These problems raise the important questions on how spatial accuracy is affected by noise in small cellular systems, what is the mechanism for controlling noise to achieve higher spatial accuracy under realistic biological conditions, and furthermore what is the free energy cost for achieving accurate pattern formation in noisy systems, which is the focus of our study.

An important function of the Turing pattern in biological systems is to enable accurate spatial positioning. For example, motivated by the Muk system for chromosome organization and DNA segregation in bacterial cells [21], Murray & Sourjik [11] proposed a simple stochastic model with three chemical species that exhibits accurate positioning of the Turing pattern, which they called ‘self-positioning’. In their model, two main chemical species representing DNA-attached and DNA-free MukBEF complex, respectively, have different diffusion constants and can convert between each other through cooperative reactions. However, even though two chemical species are enough to give rise to Turing patterns in infinite systems [5], Murray and Sourjik found that the third chemical species, which represents the dissociated Muk proteins, is crucial for self-positioning in a noisy finite system as it introduces an effective ‘long-range’ (of the order of the inter-stripe distance) interaction in the system to damp out the long length scale fluctuations (see electronic supplementary material, text and figure S1 for details).

From a statistical physics perspective, all pattern-forming systems are driven far from equilibrium and require continuous free energy dissipation for their maintenance (e.g. by ATP hydrolysis). Indeed, there are growing interests in understanding energy costs in various biological systems such as ultrasensitive biological switch [22], sensory adaptation [23], biochemical oscillation [24], biochemical error correction [25], gene regulation [26] and synchronization [27] by using standard non-equilibrium thermodynamics approach [28–30]. These studies have attracted broad interest from both theorists and experimentalists to study cellular bioenergetics, i.e. to understand how the energy cost and the corresponding ATP consumption in small biological systems, such as a single cell, can enhance or constrain the performance of biological functions, which resides at the interface of cell biology, energy metabolism and non-equilibrium physics (see Yang *et al*. [31] for a recent review). More recently, some theoretical progress has been made towards understanding stochastic thermodynamics in spatially extended systems [32–36], although most of this theoretical work so far are not directly related to realistic biological systems.

In this paper, we aim to understand the thermodynamic cost of self-positioning in realistic small reaction–diffusion systems. The paper is arranged as follows. We first describe the general non-equilibrium thermodynamics framework for spatially extended systems [32–34] based on the modified Murray–Sourjik three-species model that includes reverse reactions. We will then present our main theoretical results regarding the role of energy dissipation in enhancing the accuracy of self-positioning and the robustness of the Turing pattern against protein concentration fluctuations. Finally, we apply our theoretical framework to study a more realistic model of the MukBEF system [21]. We show the possible role of ATP hydrolysis in enhancing accuracy and robustness of the MukBEF cluster formation process and propose experiments to test predictions from our theoretical analysis.

## 2. Model and analysis

### 2.1. A reversible three-species reaction–diffusion model for self-positioned Turing pattern

**Figure 1.**
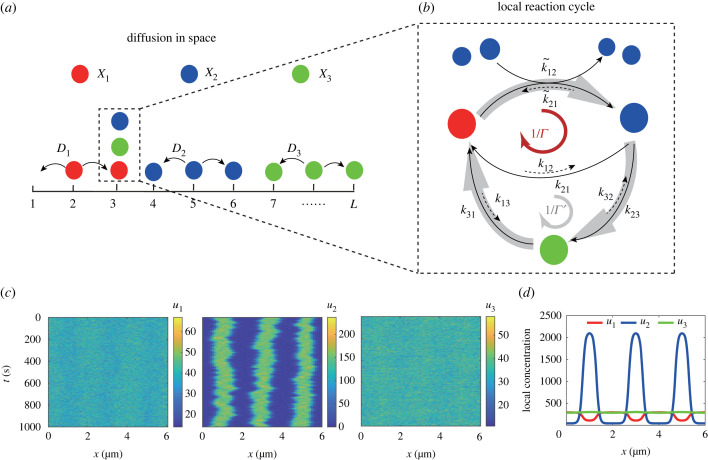
Schematic illustration of the stochastic reaction–diffusion system and its typical behaviour. (*a*) Three different bio-molecules, *X*_1_, *X*_2_ and *X*_3_, represented by different colours diffuse in physical space with different diffusion constants *D*_1,2,3_. (*b*) Within the same physical location (box) (labelled by the ‘box’ number *i* = 1, 2, …, *L*) as shown in the dotted box in (*a*), the three types of molecules interact with each other through four chemical reactions. These reactions form two reaction cycles that are characterized by their irreversibility parameters Γ and $\Gamma {\;}^{\prime }$. (*c*) The spatial–temporal plots for the concentration fields *u*_1_(*x*, *t*), *u*_2_(*x*, *t*) and *u*_3_(*x*, *t*) for *X*_1_, *X*_2_ and *X*_3_, respectively, in the Turing pattern regime. (*d*) The time averaged spatial profiles of *u*_1_, *u*_2_ and *u*_3_.

To study thermodynamics of Turing patterns in realistic biochemical systems with self-positioning, we modified the three-species reaction–diffusion model proposed by Murray & Sourjik [11] to include all reverse reactions to make it thermodynamically consistent. As shown in figure 1*a*, there are three species *X*_1_, *X*_2_, *X*_3_ representing different forms (conformations) of the same protein complex. They can convert from one form to another in four reversible reactions with different transition rates as illustrated in figure 1*b*. In addition to three ‘linear’ reactions between all pairs of species, there is a ‘nonlinear’ auto-catalytic reaction where *X*_1_ can convert to *X*_2_ in the presence of two *X*_2_

2.1 $$X_{1}\overset{k_{12}}{\underset{k_{21}}{⇌}}X_{2},\;X_{2}\overset{k_{23}}{\underset{k_{32}}{⇌}}X_{3},\;X_{3}\overset{k_{31}}{\underset{k_{13}}{⇌}}X_{1}\;\mathrm{and}\;X_{1}+2X_{2}\overset{{\tilde{k}}_{12}}{\underset{{\tilde{k}}_{21}}{⇌}}3X_{2},$$

where *k*_*ij*_ (*i* ∈ [1, 3], *j* ∈ [1, 3], *i* ≠ *j*) are reaction rates for linear conversion reactions, and ${\tilde{k}}_{12(21)}$ are rates for the reversible autocatalytic reaction. The topology of the reaction network is the same as in [11]. The key difference is that all reactions in our model are reversible with non-zero forward and backward rates, which allows us to study thermodynamics of the system properly (see electronic supplementary material for details of dynamical equations). The original Murray–Sourjik model [11] considers the irreversible limit of the autocatalytic reaction (${\tilde{k}}_{21}=0$).

The reactions given in equation (2.1), particularly the autocatalytic reaction, are similar to the Brusselator model, which is known for its ability to produce chemical oscillations. However, different from well-mixed systems, the molecules, *X*_1_, *X*_2_ and *X*_3_, can diffuse with different diffusion constants *D*_1_, *D*_2_ and *D*_3_, respectively. It was first shown by Turing [5] that when the reaction rates and the diffusion constants satisfy certain conditions, the spatially homogeneous steady-state becomes unstable (Turing instability), and the system can spontaneously form spatially inhomogeneous pattern, i.e. Turing pattern. One of the key requirements for the Turing pattern formation is that the diffusion constant of inhibitors is larger than that of activators: *d* ≡ *D*_1_/*D*_2_ > 1. In this study, we consider a realistic biological system that has a closed boundary, i.e. the system does not exchange *X*_1,2,3_ with surroundings (like proteins in a cell), which implies a no-flux boundary condition. In this study, we also assume *D*_1_ = *D*_3_, as they represent diffusion constants of the same protein but in different conformations, which are expected to have similar diffusion rates.

It is important to note that even though a two-species model can generate Turing patterns, as Turing showed in his original paper [5], the additional *X*_3_ species is crucial for positional accuracy of the Turing pattern in small noisy biological systems, as first pointed out by Murray & Sourjik [11]. More specifically, the *X*_3_ species prevents Turing patterns from being trapped in initial metastable states. Furthermore, *X*_3_ introduces an effective non-local interaction in the system at long timescale that stabilizes the Turing pattern. By contrast, the Turing pattern in the two-species Brusselator model used in [35] is more likely to be trapped in initial metastable states when the noise is small. Pattern stripes in the two-species model are also prone to merging together at long timescale. See electronic supplementary material, section V for a detailed analysis of the role of the *X*_3_ species (also see Murray & Sourjik [11] for a related discussion).

One of the main characteristics of non-equilibrium reaction networks is the existence of reaction cycles that carry persistent probability current even when the system reaches its steady state. There are two independent reaction cycles in the three-species model: *X*_1_ → *X*_2_ → *X*_1_ and *X*_1_ → *X*_2_ → *X*_3_ → *X*_1_ (see figure 1*b* for an illustration of the model). The ratios of the products of the reaction rates in the counterclockwise and clockwise directions in these two respective cycles are

2.2 $$\Gamma =\frac{{\tilde{k}}_{21}k_{12}}{{\tilde{k}}_{12}k_{21}}\;\mathrm{and}\;\Gamma {\;}^{\prime }=\frac{k_{13}k_{32}k_{21}}{k_{12}k_{23}k_{31}},$$

which characterize the irreversibility of the two reaction cycles in the three-species biochemical network as shown in figure 1*b*. The system is in equilibrium only when $\Gamma =\Gamma {\;}^{\prime }=1$. When either of these two irreversibility parameters deviates from 1, the system is out of thermal equilibrium and energy is dissipated continuously even when the system is in its steady state.

As the system is driven far from equilibrium, i.e. when Γ is lower than a critical value, the spatial homogeneity is spontaneously broken and the Turing pattern emerges. The spatial–temporal dynamics of the three-species model is studied numerically (see electronic supplementary material for the technical details of simulations). A typical Turing pattern in our system and its time-averaged profiles from our simulations are shown in figure 1*c*,*d*. Next, we consider the energy cost of the reaction diffusion system underlying the Turing pattern.

### 2.2. Dissipation in the reversible three-species reaction–diffusion model

All pattern forming reaction–diffusion systems are out of equilibrium [32,37]. Continuous energy dissipation is needed to maintain the non-equilibrium steady state (NESS) with the energy sources to sustain reactant gradients in chemical reaction systems [38] or continuous ATP hydrolysis in biological systems. By following the same procedure as in previous work [32–34], we briefly describe how we compute the NESS dissipation rate in the three-species reaction–diffusion model.

For a spatially extended system, the free energy dissipation rate consists of two parts: the first part is due to local chemical reactions and the second part corresponds to energy dissipation for maintaining non-uniform concentration field. Owing to the spatial dependence of the concentration fields, we compute the energy dissipation rate per unit length for one-dimensional system studied here with the general definition of dissipation rate ${\dot{W}}_{\mathrm{individual}}$ for each individual reaction [29],

2.3 $${\dot{W}}_{\mathrm{individual}}=(J^{+}-J^{-})\ln\left(\frac{J^{+}}{J^{-}}\right),$$

where *J*^+^ and *J*^−^ are forward and backward fluxes, respectively, between two microscopic states.

For the chemical reactions, the local dissipation rate *density* $\dot{w}(x)$ at position *x* can be computed the same way as in homogeneous systems

2.4 $${\dot{w}}_{\mathrm{chem}}(x)=\sum_{i=1}^{N_{r}}[j_{i}^{+}(x)-j_{i}^{-}(x)]\ln\left(\frac{\;j_{i}^{+}(x)}{j_{i}^{-}(x)}\right),$$

where *N*_*r*_ = 4 is the number of reactions in the biochemical network and $j_{i}^{+}(x)$ and $j_{i}^{-}(x)$ are the forward and backward flux *densities* of the *i*th reaction at position *x*, and free energy is in units of thermal energy (*k*_*B*_*T*). For the reaction between *X*_2_ and *X*_3_, we have: *j*^+^(*x*) = *k*_23_ *u*_2_(*x*), *j*^−^(*x*) = *k*_32_*u*_3_(*x*), with *u*_*i*_(*x*) the local concentrations of molecules *X*_*i*_.

The dissipation due to transport in space such as diffusion can be calculated by considering the spatial degrees of freedom as state variables. We divide the space into small boxes with size Δ*x*, the dissipation rate of the free energy density ${\dot{w}}_{\mathrm{diff}}(x)$ due to diffusion between neighbouring boxes can be obtained by considering the diffusive transport fluxes as the forward and backward fluxes in the extended state-space. In particular, the forward and backward diffusive fluxes for the molecule *X*_*k*_ are $J_{D,k}^{+}(x)=u_{k}(x)\times {\tilde{D}}_{k}\Delta x$ and $J_{D,k}^{-}(x)=u_{k}(x+\Delta x)\times {\tilde{D}}_{k}\Delta x$, where ${\tilde{D}}_{k}$ is microscopic transition rate scaled from diffusion rate, ${\tilde{D}}_{k}\equiv D_{k}/\Delta x^{2}$. Plugging these two fluxes into equation (2.3), we have

2.5 $${\dot{w}}_{\mathrm{diff}}(x)=\sum_{k=1}^{3}\frac{D_{k}{\left(\partial u_{k}/\partial x\right)}^{2}}{u_{k}(x)},$$

where *u*_*k*_(*x*) is the local concentration of *X*_*k*_ molecule and the summation goes over all species (*k* = 1, 2, 3).

The total energy dissipation rate $\dot{W}$ for the whole system is the sum of these two dissipation rate densities ${\dot{w}}_{\mathrm{chem}}(x)$ and ${\dot{w}}_{\mathrm{diff}}(x)$ integrated over space. In steady state, the net fluxes of reaction and diffusion should balance each other for all molecule species. Using these steady-state conditions, we can drastically simplify the expression for the total dissipation rate (see electronic supplementary material for details),

2.6 $$\begin{array}{rl} \dot{W} & =\int ({\dot{w}}_{\mathrm{diff}}+{\dot{w}}_{\mathrm{chem}})\;dx=\sum_{i=1}^{N_{r}}\ln\left(\frac{k_{i}^{+}}{k_{i}^{-}}\right)\int_{0}^{L}[j_{i}^{+}(x)-j_{i}^{-}(x)]\;dx\\ & =J_{c1}\ln\Gamma^{-1}+J_{c2}\ln\Gamma {\;}^{\prime -1}, \end{array}$$

where $k_{i}^{+}$ and $k_{i}^{-}$ are the forward and backward reaction rate constants for the *i*th chemical reaction, and *J*_1*c*_ and *J*_2*c*_ are the total fluxes in the two cycles with irreversible parameters Γ and $\Gamma^{\prime }$, which can be expressed as $J_{1c}=\int_{0}^{L}(k_{12}u_{1}$ $(x)-k_{21}u_{2}(x))dx=k_{12}N_{1}-k_{21}N_{2}$, $J_{2c}=\int_{0}^{L}(k_{23}u_{2}(x)-k_{32}u_{3}(x))\;$ $dx=k_{23}N_{2}-k_{32}N_{3}$, where *N*_*i*_ is the total number of *X*_*i*_ molecules in the system. Plugging these expressions in equation (2.6), we arrive at a surprisingly simple equation for the expression for the total dissipation rate

2.7 $$\begin{array}{r} \dot{W}=(k_{12}N_{1}-k_{21}N_{2})\ln\left(\frac{1}{\Gamma }\right)+(k_{23}N_{2}-k_{32}N_{3})\ln\left(\frac{1}{\Gamma^{\prime }}\right), \end{array}$$

which does not depend on the diffusion constants explicitly. This result makes intuitive sense as diffusion by itself is not an active process, and it only affects dissipation when chemical concentrations *u*_*k*_(*x*) become spatially non-uniform and additional energy is needed to overcome diffusion to maintain the spatial pattern.

## 3. Results

### 3.1. Dependence of dissipation on chemical driving force and diffusion constant ratio

In the three-species model, the *X*_3_-related kinetic rates, i.e. *k*_13_, *k*_23_, *k*_32_ and *k*_31_ are much smaller than the other rate constants. Therefore, the energy dissipation is spent predominately in driving the Γ cycle. In this study, we vary Γ by changing the reverse autocatalytic reaction rate ${\tilde{k}}_{21}$ while keeping other kinetic rates fixed. We define W≡−ln⁡(Γ) to measure the dominant chemical driving force. Besides reaction rates, the Turing pattern depends critically on the diffusion constant ratio *d*: only when *d* is larger than a critical value *d*_*c*_, the spatially homogeneous steady state can become unstable. Here, we study pattern formation and its energy dissipation rate ($\dot{W}$) in the parameter regime beyond the Turing instability, i.e. where the spatially homogeneous state is unstable due to Turing instability in the finite system. It is worth mentioning that ‘quasi-pattern’ driven by intrinsic noise could emerge in regions of parameter space before the onset of the Turing instability where the homogeneous state is linearly stable in the deterministic model (infinite system) [12–15]. The mechanism for stochastic ‘quasi-pattern’ formation is different from the original Turing mechanism and we leave the energetics of the noise-driven Turing patterns for future study.

**Figure 2.**
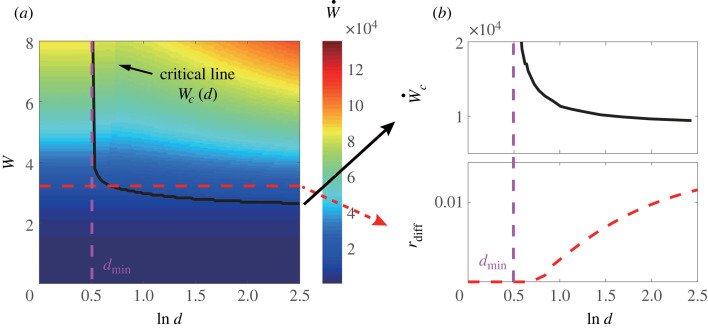
Onset of the Turing pattern and its energy cost. (*a*) The dependence of the energy dissipation rate ($\dot{W}$) on the chemical driving force (*W*) and the diffusion constant ratio (*d*). The black line represents the critical line (*W*_*c*_(*d*)) for the onset of Turing pattern. The vertical purple dotted line shows the minimum value of *d*, *d*_min_, below which no Turing pattern is possible, independent of the chemical driving force *W*. (*b*) The critical energy dissipation rate ${\dot{W}}_{c}\equiv \dot{W}(W_{c}(d),d)$ (solid black line) versus *d*. When *d* < *d*_min_, Turing pattern does not exist. For *d* > *d*_min_, ${\dot{W}}_{c}$ decreases with *d* but saturates to a finite value when *d* → ∞. The red dotted line shows the fraction of energy dissipation due to diffusion $r_{\mathrm{diff}}=\int {\dot{w}}_{\mathrm{diff}}\;dx/\dot{W}$ versus *d* for a fixed *W* = 3.21, which corresponds to the red dotted line in (*a*). *r*_diff_ = 0 in homogeneous state when *W* < *W*_*c*_(*d*), and increases with *d* in the Turing regime but saturates to a small value at *d* → ∞.

In figure 2*a*, we show the dependence of energy dissipation rate $\dot{W}$ on the chemical driving force *W* and ln(*d*). The transition from homogeneous state (no pattern) to a three-stripe Turing pattern is shown by the solid line in figure 2*a*. We find that the onset of pattern formation occurs as the chemical driving force becomes larger than a critical value *W*_*c*_(*d*), which decreases with *d*. However, even in the limit *d* → ∞, *W*_*c*_ remains finite. The finite *W*_*c*_ for all values of *d* means a finite critical energy dissipation rate ${\dot{W}}_{c}$ is needed to generate and maintain the spatial pattern. On the other hand, when *d* is less than a minimum value *d*_min_ ≈ 1.7, no pattern formation is possible even with an infinite chemical driving force, as is shown in figure 2*b*. Here, the onset of Turing pattern and the values of *d*_min_ and *W*_*c*_(*d*) are estimated by using simulations and linear stability analysis of the deterministic equations in finite systems, which are consistent with the averaged results from direct numerical simulations of the stochastic reaction–diffusion processes.

The overall dissipation rate consists of two parts: the dissipation in the chemical reactions and the dissipation used to overcome the diffusion. We define the fraction of the energy dissipation used to overcome diffusion as

3.1 $$r_{\mathrm{diff}}\equiv \frac{\int {\dot{w}}_{\mathrm{diff}}\;dx}{\int ({\dot{w}}_{\mathrm{diff}}+{\dot{w}}_{\mathrm{chem}})\;dx}.$$

In the parameter regime where spatially homogeneous state is stable, i.e. before the onset of Turing pattern, the concentration fields are spatially uniform and there is no dissipation to overcome diffusion: *r*_diff_ = 0. When the chemical driving force *W* is larger than the critical value *W*_*c*_(*d*), the Turing pattern emerges, and the fraction of energy dissipated to overcome the diffusion in order to maintain the spatially inhomogeneous pattern becomes non-zero: *r*_diff_ > 0. The dependence of *r*_diff_ on diffusion constant ratio *d* with a fixed chemical driving force *W* is shown in figure 2*b* (red dotted line). As expected, the diffusion dissipation ratio *r*_diff_ increases with *d* when *d* > *d*_*c*_(*W*), where *d*_*c*_(*W*) is the critical diffusion constant ratio at a given *W*. Note that *d*_*c*_(*W*) decreases with increasing chemical driving force *W* and it approaches its minimum value *d*_min_ when chemical driving force approaches infinity, i.e. *d*_min_ = *d*_*c*_(*W* = ∞) (the purple dotted line in figure 2*a*,*b*). Deep in the Turing pattern regime, in the limit *d*, *W* ≫ 1, the ratio between the two dissipation rates can be estimated as (see electronic supplementary material, section IV for details)

3.2 $$\frac{\int {\dot{w}}_{\mathrm{diff}}\;dx}{\int {\dot{w}}_{\mathrm{chem}}\;dx}\approx \frac{2}{\pi^{2}W}{\left(\frac{\Delta u_{2}}{\left\langle u_{2}\right\rangle }\right)}^{2},$$

where Δ*u*_2_ = (*u*_2,max_ − *u*_2,min_)/2 is the amplitude of the spatial variation in *u*_2_(*x*). *u*_2,max_ and *u*_2,min_ are maximum and minimum value of *u*_2_(*x*). 〈*u*_2_〉 is the average of *u*_2_(*x*) over space.

Overall, most of the energy is dissipated to drive chemical reactions to generate the Turing instability. After the onset of Turing pattern, the fraction of energy used for maintaining the spatial gradients against the diffusion becomes non-zero, and it increases as the relative amplitude of the Turing pattern increases. However, *r*_diff_ remains small even deep in the Turing pattern regime.

### 3.2. The error–energy relation for self-positioned Turing pattern in small systems

**Figure 3.**
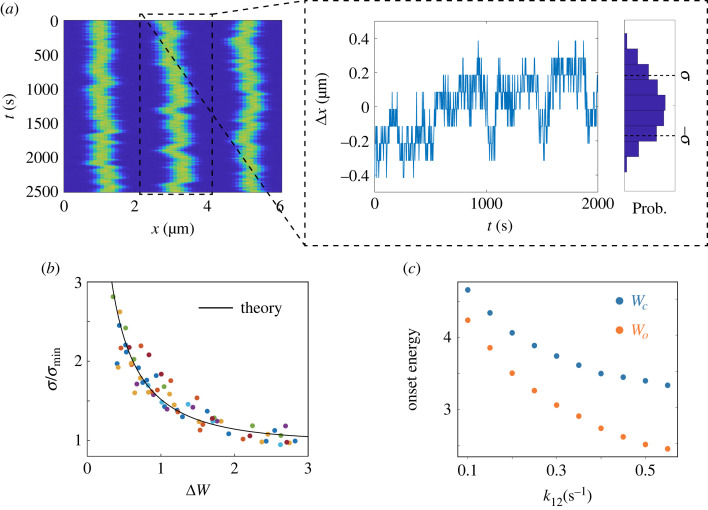
The accuracy–energy relationship in self-positioned Turing pattern. (*a*) The spatial–temporal profile (kymograph) of *u*_2_(*x*, *t*). The fluctuations of the peak position *x*_*p*_(*t*) of the central stripe is shown in the dotted box. Δ*x* = *x*_*p*_(*t*) − 〈*x*_*p*_〉_*t*_ is the deviation of the peak position from its mean at time *t*. The distribution of Δ*x* with a variance *σ* is also shown. (*b*) The positional error *σ*/*σ*_min_ versus the free-energy dissipation per cycle in additional to the critical energy, Δ*W*. Δ*W* is varied by changing ${\tilde{k}}_{21}$ for different values of *k*_12_ = 0.1, 0.15, 0.2, 0.25, 0.3, 0.35, 0.4, 0.45, 0.5, 0.55 s^−1^, which are represented by different colours. All data for different choices of *k*_12_ and ${\tilde{k}}_{21}$ collapse onto the same curve that can be fitted by our theoretical prediction, equation (3.8), with fitted parameters: *c*_1_ = 0.93 and *c*_*i*_ = 0 for *i* ≥ 2 (solid line). (*c*) The onset energy for finite system (*W*_*c*_) and infinite system (*W*_0_) versus *k*_12_. It is clear that *W*_*c*_ > *W*_0_ and both increases as *k*_12_ decreases. Other parameters used are: ${\tilde{k}}_{12}=1.67\times 10^{-5}\;s^{-1}\;\mu m^{2}$, *k*_21_ = 3.6 s^−1^, *k*_13_ = *k*_23_ = 0.0139 s^−1^, *k*_31_ = 0.0416 s^−1^, *k*_32_ = 1.39 × 10^−5^ s^−1^, *D*_1_ = *D*_3_ = 0.3 μm^2^ s^−1^, *D*_2_ = 0.012 μm^2^ s^−1^. The length of the system is *L* = 6 μm. In our stochastic simulations, the space is divided into 60 ‘boxes’ with the box size Δ*x* = 0.1 μm (see electronic supplementary material for details). Total number of molecules *N* = 7200.

Turing patterns spontaneously break the spatial translation symmetry of the underlying homogeneous biochemical reaction–diffusion system. As a result, the ‘phase’ degree of freedom of the Turing pattern is a soft mode that can have large fluctuations due to noise in finite biochemical systems with a small number of molecules. In figure 3*a*, a time series of the peak location (*x*_*p*_(*t*)) for one of the molecular species *X*_2_ is shown. The standard deviation (*σ*) of *x*_*p*_, $\sigma \equiv \sqrt{{\left\langle {\left(x_{\;p}(t)-{\bar{x}}_{\;p}\right)}^{2}\right\rangle }_{t}}$, can be used as a measure of the spatial error of the Turing pattern.

In an infinite system, the most unstable mode has a wavevector *q*_0_, which is the wavevector with the highest linear growth rate *ρ*(*q*), i.e. $(d\rho /dq){|}_{q_{0}}=0$. In a finite system with size *L*, the Turing pattern wavevector has the form, *q*_*n*_ = 2*π*/*λ*_*n*_ where *λ*_*n*_ = *L*/*n* (*n* ≥ 1, n∈Z) is the wavelength. Typically, the wavevector in a finite system is different from the one in an infinite system: *q*_*n*_ ≠ *q*_0_ and their difference is given by Δ*q* ≡ *q*_*n*_ − *q*_0_( ≠ 0). The spatial–temporal profile of a concentration field (e.g. *u*_2_(*x*, *t*)) in a Turing pattern can be written as

3.3 $$u_{2}(x,t)=\Theta (q_{n}x+\phi (x,t)),$$

where Θ is a periodic function with period 2*π* and *ϕ*(*x*, *t*) is the phase variable of the Turing pattern. The phase variable satisfied the phase diffusion equation [39], which can be generally written as

3.4 $$\frac{\partial \phi }{\partial t}=D_{2}\frac{\partial^{2}\phi }{\partial x^{2}}-D_{4}\frac{\partial^{4}\phi }{\partial x^{4}}+\partial_{x}\eta ,$$

where *D*_2_ is the second-order diffusion term, and a fourth-order diffusion term with *D*_4_ > 0 is introduced to prevent divergence when *D*_2_ → 0. The form of the noise term ∂_*x*_*η* is due to the translational invariance of the phase variable *ϕ* and *η* is a Gaussian white noise: 〈*η*(*x*, *t*)*η*(*x*′, *t*′)〉 = Δ_0_*δ*(*t* − *t*′)*δ*(*x* − *x*′) with Δ_0_ the noise strength.

Following the standard procedure [39], the second-order phase diffusion constant *D*_2_ can be expressed as

3.5 $$D_{2}=\xi^{2}\tau^{-1}\frac{\epsilon_{0}-3\xi^{2}\Delta q^{2}}{\epsilon_{0}-\xi^{2}\Delta q^{2}},$$

where the control parameter is defined as $\epsilon_{0}\equiv 1-{\tilde{k}}_{21}/{\tilde{k}}_{0}$ with ${\tilde{k}}_{0}$ the critical value of ${\tilde{k}}_{21}$ in an infinite system. *ξ* is a characteristic length given by $\xi^{2}=-\frac{1}{2}(d^{2}\rho /dq^{2}){|}_{q_{0}}$, and *τ* is a characteristic timescale. In an infinite system, a Turing pattern appears when $\epsilon_{0}\geq 0$ or equivalently ${\tilde{k}}_{21}\leq {\tilde{k}}_{0}$. In a finite system, however, the requirement for a Turing pattern with phase stability (*D*_2_ > 0) becomes more stringent due to the non-zero Δ*q*(≠ 0). For a stable Turing pattern with the wavevector *q*_*n*_ in a finite system, the critical value ${\tilde{k}}_{c}$ of ${\tilde{k}}_{21}$ can be defined when the phase diffusion constant becomes zero, i.e. *D*_2_ = 0. From equation (3.5), we determine the critical value ${\tilde{k}}_{c}={\tilde{k}}_{0}(1-3\xi^{2}\Delta q^{2})<{\tilde{k}}_{0}$, which represents a stronger requirement than that in the infinite system.^1^ Based on this critical value ${\tilde{k}}_{c}$, a new control parameter can be defined as $\epsilon \equiv 1-{\tilde{k}}_{21}/{\tilde{k}}_{c}$ for a finite system, describing the distance in parameter space from the onset of the Turing pattern. The phase diffusion constant $D_{2}(\epsilon )$ is an increasing function of ϵ with $D_{2}(\epsilon =0)=0$. From ${\tilde{k}}_{c}$, we can also define a critical value $\Gamma_{c}\equiv k_{12}k_{c}/k_{21}{\tilde{k}}_{12}$ for Γ in finite systems, so $\epsilon =1-\Gamma /\Gamma_{c}$. Note that $\Gamma_{c}<\Gamma_{0}$ because ${\tilde{k}}_{c}<{\tilde{k}}_{0}$, which means the phase stability of the Turing pattern in a finite system requires a higher dissipation rate (per molecule) than the onset energy $W_{0}(\equiv -\ln(\Gamma_{0}))$ in the infinite system.

From the stochastic phase equation (equation 3.4), we can compute the positional variance *σ*^2^, which is proportional to the phase variance,

3.6 $$\begin{array}{rl} \sigma^{2} & \equiv {\left(\frac{\lambda }{2\pi }\right)}^{2}\langle \phi^{2}\rangle \\ & ={\left(\frac{\lambda }{2\pi }\right)}^{2}\int_{\pi /L}^{\infty }\int_{-\infty }^{\infty }\frac{\Delta_{0}q^{2}\;d\omega dq}{\omega^{2}+{\left(D_{2}(\epsilon )q^{2}+D_{4}q^{4}\right)}^{2}}=\frac{\sigma_{0}^{2}}{S(\epsilon )}, \end{array}$$

where *ω* and *q* represent the frequency and wavevector, respectively; $\sigma_{0}^{2}$ is the position variance when ϵ=0 ($\Gamma =\Gamma_{c}$). S(ϵ) is the variance reduction factor, which is an increasing function of ϵ with *S*(0) = 1. Given that *D*_2_ = 0 when ϵ=0, we can use a linear approximation for $D_{2}=d_{2}\epsilon$ with a positive constant *d*_2_( > 0). By further assuming a constant *D*_4_ > 0 in equation (3.6), we have $\sigma_{0}^{2}={\left(\lambda /2\pi \right)}^{2}\Delta_{0}L/D_{4}$ and $S(\epsilon )=a\epsilon^{1/2}/\tan^{-1}(a\epsilon^{1/2})$ with a constant *a* = (*L*/*π*)(*d*_2_/*D*_4_)^1/2^.

Equation (3.6) clearly shows that the positional error *σ* decreases as $\epsilon =1-{\tilde{k}}_{21}/{\tilde{k}}_{n}=1-\Gamma /\Gamma_{c}$ increases or equivalently when Γ decreases. According to equation (2.6), the total energy dissipation can be decomposed into those from each cycle: $\dot{W}={\dot{W}}_{1}+{\dot{W}}_{2}=J_{1c}\ln(\Gamma^{-1})+J_{2c}\ln(\Gamma^{\prime -1})$, where *J*_1*c*_ and *J*_2*c*_ are the fluxes of the two cycles integrated over space. In the three-species model, the energy dissipation is dominated by the first cycle as the flux in the first cycle is much larger than that in the second cycle: *J*_1*c*_ ≫ *J*_2*c*_ or equivalently the cycle time $\tau_{1}\equiv J_{1c}^{-1}$ for the first cycle is much shorter than that of the second cycle $\tau_{2}\equiv J_{2c}^{-1}$: *τ*_1_ ≪ *τ*_2_. As a result, the total energy dissipation per molecule during the dominant cycle time *τ*_1_ is $\ln(\Gamma^{-1})+(\tau_{1}/\tau_{2})\ln(\Gamma^{\prime -1})\approx \ln(\Gamma^{-1})=W$, which is approximately the chemical driving force defined before. We can relate energy dissipation per molecule *W* with the control parameter ϵ defined above,

3.7 $$\epsilon =1-\frac{\Gamma }{\Gamma_{c}}\approx 1-\exp(W_{c}-W)\equiv 1-\exp(-\Delta W),$$

where $W_{c}\equiv \ln(\Gamma_{c}^{-1})$ denotes the critical (onset) energy dissipation per cycle in the finite system and Δ*W* ≡ *W* − *W*_*c*_ is the additional energy dissipation per cycle beyond the critical energy dissipation *W*_*c*_.

In general, the system is most stable in the limit of ϵ→1, i.e. the strong driving limit Δ*W* → ∞, where the error $\sigma (\epsilon =1)\equiv \sigma_{\min}=\sigma_{0}/\sqrt{S(1)}$ is at its minimum. From equation (3.6), we can write $\sigma (\epsilon )=\sigma_{\min}/r(\epsilon )$ with an error reduction function $r(\epsilon )\equiv {\left(S(\epsilon )/S(1)\right)}^{1/2}$. Since S(ϵ) is an increasing function of ϵ, r(ϵ) is also an increasing function of ϵ with *r*(1) = 1. In the strong driving (or high dissipation) limit, we can expand r(ϵ) around ϵ=1: $r(\epsilon )=1+\sum_{i=1}c_{i}{\left(\epsilon -1\right)}^{i}$ with constant coefficients *c*_*i*_ (*c*_1_ > 0). From equation (3.6) and by using the dependence of ϵ on Δ*W*, we obtain the error–energy relation

3.8 $$\sigma =\frac{\sigma_{\min}}{r(\epsilon )}=\frac{\sigma_{\min}}{1-c_{1}\exp(-\Delta W)+h.o.t.},$$

where only the first leading-order terms (*c*_1_) is written out explicitly for simplicity (h.o.t. stands for higher order terms). Equation (3.8) clearly shows that positional error can be suppressed by increasing energy dissipation.

We have tested this error–energy dependence (equation (3.8)) by extensive simulations of the three-species model for different values of ${\tilde{k}}_{21}$ and *k*_12_ using Gillespie algorithm [40]. As shown in figure 3*b*, the dependence of the normalized positional error *σ*/*σ*_min_ on the additional energy dissipation Δ*W* collapsed onto the same curve that can be fitted by equation (3.8) for all different values of ${\tilde{k}}_{21}$ and *k*_12_. The dependence of *W*_0_ and *W*_*c*_ on *k*_12_ are shown in figure 3*c*, which clearly shows that the critical energy for a finite system is larger than that for the infinite system: *W*_*c*_ > *W*_0_ and both decrease with *k*_12_. Both the analytical and numerical results clearly show that a larger dissipation per cycle Δ*W* (or equivalently a smaller Γ) suppresses the phase fluctuations and leads to a higher positional accuracy in the Turing pattern.

The monotonic decrease and saturation of positional error with increasing dissipation rate in the three-species model as shown in figure 3*b* and equation (3.8) is notably different from the non-monotonic dependence found in a two-species Brusselator model [35]. The explanation is that without the self-positioning mechanism enabled by the additional *X*_3_ chemical species, Turing patterns can be trapped in metastable states, which could lead to the increase of spatial error in the small fluctuation or large dissipation limit [35]. By contrast, the additional *X*_3_ species in the three-species model help the system escape the initial metastable states. Furthermore, the slowly varying *X*_3_ introduces an effective non-local interaction in the system at long timescale to stabilize the Turing pattern (see electronic supplementary material, section V for details). The monotonic error–energy relation should hold as long as there are some slow stabilizing components in the system, as we will show later in a more complex model for the Muk system. Given the importance of accurate pattern positioning in biology, we expect the existence of the ‘stabilizer’ species *X*_3_ to be general in realistic cellular processes.

### 3.3. Free energy dissipation enhances the robustness of Turing pattern against concentration fluctuations

**Figure 4.**
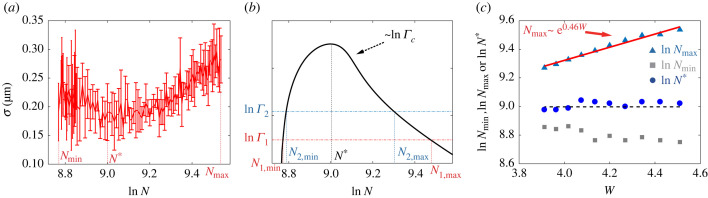
Dependence of Turing pattern on total molecule number. (*a*) Positional error *σ* has a U-shape dependence on the molecule number *N*. The three-stripe Turing pattern is stable in a finite range *N*_min_ ≤ *N* ≤ *N*_max_ with *σ* reaching its minimum at N*. (*b*) The non-monotonic dependence of $\Gamma_{c}$ on *N*. Two different choices of Γ are shown to illustrate how *N*_min_, *N*_max_ and N* should vary with Γ. (*c*) The dependence of *N*_min_, *N*_max_ and N* on *W* (energy dissipation per cycle). A linear fit between ln *N*_max_ and *W* (red line) has a slope 0.46, which is close to the theoretical value 0.5 (equation 3.11). N* is independent of *W* and close to the maximum position of $\Gamma_{c}(N)$ (black dotted line). Parameters used here are Γ=0.011, ${\tilde{k}}_{21}=1.67\times 10^{-5}\;s^{-1}\;\mu m^{2}$, *k*_12_ = 0.5 s^−1^, *k*_21_ = 3.6 s^−1^, *k*_13_ = *k*_23_ = 0.139 s^−1^, *k*_31_ = 0.416 s^−1^, *k*_32_ = 0.0139 s^−1^, *D*_1_ = *D*_3_ = 1.8 μm^2^ s^−1^, *D*_2_ = 0.012 μm^2^ s^−1^.

In small biological systems such as a bacterial cell, the size of the system is tightly regulated but the protein concentration can fluctuate in time and vary from cell to cell [41–46]. Here, we study how the positional error *σ* depends on the molecule (protein) concentration by varying the total molecule number *N* in the three-species model while keeping the length of the system *L* fixed. Intuitively, since the overall noise level (fluctuation) scales as *N*^−1/2^, increasing *N* is expected to lead to a higher spatial accuracy. However, in a biochemical reaction system with nonlinear reaction dynamics, increasing *N* also affects the system’s sensitivity to fluctuations, which makes the overall effect of varying *N* on spatial accuracy unclear. From our simulation results, we found that a specific spatial pattern (e.g. a stable three-stripe pattern) only exists in a finite range of molecule copy number: *N*_min_ < *N* < *N*_max_. The system transitions to other spatial patterns (e.g. two-stripe patterns) when *N* is outside of this range. As shown in figure 4*a*, the dependence of *σ* on *N* (for *N*_min_ < *N* < *N*_max_) follows a non-monotonic ‘U’-shape and there exists an optimal molecule number N* where the positional error *σ* is minimal. More specifically, *σ* does decrease as *N* increases when *N*_min_ < *N* < N*; however, for N* < *N* < *N*_max_, the positional error *σ* increases as *N* increases, which is counterintuitive.

How does this non-monotonic dependence of *σ* on *N* arise? As shown before, the positional error of the Turing pattern can be written as $\sigma =\sigma_{\min}r^{-1}(\epsilon )\propto \Delta^{1/2}r^{-1}(\epsilon )$ where the overall noise intensity Δ is inversely proportional to the total molecule number Δ ∝ *N*^−1^ and the inverse noise reduction factor $r^{-1}(\epsilon )$ can be understood as the sensitivity (susceptibility) to noise. To understand the *N*-dependence, we define the relative concentrations *v*_*i*_ ≡ *u*_*i*_/*c*_tot_ (*i* = 1, 2, 3) with *c*_tot_ = *N*/*L* the total molecule concentration. Dynamics of the relative concentrations *v*_*i*_ are governed by the same equations as those for *u*_*i*_ but with effective reaction rates. For the linear reactions, the effective reaction rates remain the same as the original rates. However, for the nonlinear reactions, e.g. the autocatalytic reaction, the effective reaction rates are normalized by *c*_tot_: $\beta_{12(21)}={\tilde{k}}_{12(21)}c_{\mathrm{tot}}^{2}={\tilde{k}}_{12(21)}N^{2}/L^{2}$ (note that ${\tilde{k}}_{12(21)}$ are the intrinsic reaction rates and are independent of concentration). Thus, the noise susceptibility $r^{-1}(\epsilon )$ depends on *N* because the control parameter $\epsilon \equiv 1-\Gamma /\Gamma_{c}$ depends on the critical value $\Gamma_{c}$, which depends on *N* through its dependence on *β*_12_ and *β*_21_.

As described earlier in this paper, the critical reaction rate (${\tilde{k}}_{c}$) for a stable three-stripe pattern is proportional to the onset reaction rate (${\tilde{k}}_{0}$) in an infinite system: ${\tilde{k}}_{c}=\zeta {\tilde{k}}_{0}$ with *ζ* = 1 − 3*ξ*^2^Δ*q*^2^ approximately a constant. Therefore, we have $\Gamma_{c}\equiv {\tilde{k}}_{c}k_{12}/{\tilde{k}}_{12}k_{21}=\zeta {\tilde{k}}_{0}k_{12}/{\tilde{k}}_{12}k_{21}\equiv \zeta \Gamma_{0}$ where $\Gamma_{0}$ can be expressed as

3.9 $$\Gamma_{0}\equiv \frac{\beta_{0}k_{12}}{\beta_{12}k_{21}},$$

with *β*_0_ the critical effective rate of *β*_21_, which can be determined by the linear stability analysis of the dynamic equations for *v*_*i*_ (see electronic supplementary material, section III for details). In the limit *d* ≫ 1, we have

3.10 $$\beta_{0}=-2\frac{k_{12}}{R_{1}}\frac{1}{v_{2}^{*3}}+\left(2k_{12}\frac{R_{2}}{R_{1}}+k_{21}\right)\frac{1}{v_{2}^{*2}},$$

where *R*_1_ = (*k*_31_ + *k*_32_ + *k*_13_)/(*k*_31_ + *k*_32_) > 1 and *R*_2_ = (*k*_31_ + *k*_32_ + *k*_23_)/(*k*_31_ + *k*_32_) > 1 are two constants, and $v_{2}^{*}$ is the relative concentration of the homogeneous fixed point solution, which depends on *N* via its dependence on *β*_12_ and *β*_21_. As *N* increases, the nonlinear autocatalytic reaction becomes more dominant as both *β*_12_ and *β*_21_ increase with *N*. For typical kinetic rates with *k*_12_ ≪ *k*_21_ and ${\tilde{k}}_{12}\gg {\tilde{k}}_{21}$ as used in our model, the dominance of the autocatalytic reaction at larger *N* leads to a higher value of $v_{2}^{*}$, i.e. $v_{2}^{*}$ increases with *N*. Finally, since the cubic and quadratic terms in equation (3.10) have opposite signs, *β*_0_ and therefore $\Gamma_{0}$ can be a non-monotonic function of $v_{2}^{*}$ and consequently a non-monotonic function of *N*.

By using equations (3.9) and (3.10), we can compute the dependence of $\Gamma_{c}(N)=\zeta \Gamma_{0}(N)$ on *N* numerically. As shown in figure 4*b*, as *N* increases, $\Gamma_{c}$ first rises sharply to a peak at N* before decreasing more gradually. For given values of reaction rates, the range of *N* for the three-stripe Turing pattern is set by $\Gamma_{c}(N)=\Gamma (\equiv {\tilde{k}}_{21}k_{12}/{\tilde{k}}_{12}k_{21})$, which determines the minimum and maximum molecule number *N*_min_ and *N*_max_. The non-monotonic dependence of $\Gamma_{c}(N)$ on *N* explains the origin of the U-shaped dependence of the positional error (*σ*) on the total number of molecules (*N*) and the finite range of *N* for the existence of the Turing pattern in a system with a fixed size as observed in figure 4*a*.

It is clear from our analysis and figure 4*b* that both *N*_min_ and *N*_max_ change with Γ or equivalently the energy dissipation of the system W≡−ln⁡Γ, whereas the optimal molecule number N* is independent of *W*. With an increased dissipation *W* (by decreasing Γ), *N*_min_ decreases and *N*_max_ increases, both of which broaden the range defined by *R* ≡ *N*_max_/*N*_min_. Since the dependence of $\Gamma_{c}(N)$ on *N* has a sharp rise and a more gradual decay, *N*_max_ is more sensitive to the change of *W* than *N*_min_. To test this result, we determined *N*_max_, *N*_min_ and N* in our simulations for different values of Γ or *W*. As shown in figure 4*c*, *N*_max_ increases with *W* whereas *N*_min_ decreases albeit weakly with *W*. N* almost keeps constant near the maximum position of $\Gamma_{c}(N)$. In the limit of large dissipation when Γ≪1, $v_{2}^{*}$ will saturate for large *N* and so will *β*_0_, so the dependence of $\Gamma_{c}$ on *N* is dominated by the factor $\beta_{12}^{-1}\propto N^{-2}$. As a result, we have (see electronic supplementary material for details) $\Gamma_{c}\approx \tilde{\alpha }N^{-2},$ where $\tilde{\alpha }$ is a coefficient depending on model parameters. By using this asymptotic behaviour of $\Gamma_{c}(N)$, we can solve $\Gamma_{c}(N_{\max})=\Gamma$ and obtain

3.11 $$N_{\max}\approx {\left(\frac{\tilde{\alpha }}{\Gamma }\right)}^{1/2}\propto \;e^{W/2}.$$

Equation (3.11) shows that *N*_max_ increases with *W* exponentially, which is confirmed by numerical results shown in figure 4*c*. The steep increase of $\Gamma_{c}$ near *N*_min_ indicates a relatively weak decrease of *N*_min_ with *W*, which is also consistent with numerical results shown in figure 4*c*. Note that (3.11) is derived under the condition *d* ≪ 1. For a finite *d* and very small $\Gamma \ll d^{-1}$, *N*_max_ saturates to a value controlled by *d*^−1^ (see electronic supplementary material for detailed discussion).

Overall, our results show that there is a finite range of concentrations over which a specific Turing pattern is stable due to nonlinearity in the reaction kinetics. A higher dissipation *W* can broaden this range, which enhances the robustness of the desired Turing pattern against the inevitable concentration variations in living cells.

### 3.4. A realistic biological system

Finally, we study the role of energy dissipation by considering a realistic biochemical system that achieves spatial positioning via the self-positioning Turing mechanism, namely the Muk system [11,21] responsible for DNA segregation in *E. coli*.

A MukBEF complex consists of three types of proteins: a MukB dimer, which is a distant relative of structural maintenance of chromosomes (SMC) protein family and is the core of the MukBEF complex, and two small accessory proteins MukE and MukF. The MukB dimer has a rod-and-hinge structure, which forms a loop to capture DNA. It also has an ATP binding domain, and experiments show that the MukB dimer serves as an ATP-dependent ‘DNA binding switch’: ATP binding promotes attachment of the MukBEF complex to DNA, whereas hydrolysis of the bound ATP stimulates DNA detachment.

**Figure 5.**
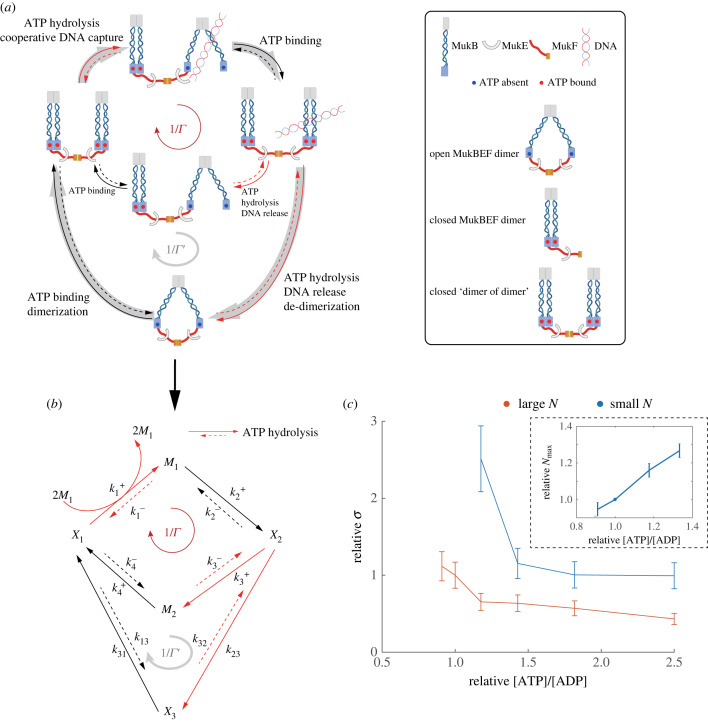
A model of Muk system for DNA segregation. (*a*) Illustration of the Muk system. There are two cycles (red and grey) in our system with the red cycle controlling the pattern’s positional order. (*b*) The corresponding reaction network. Red lines indicate the reactions involving ATP hydrolysis. The ratio of the forward reaction rate and the backward reaction rate of these ATP-hydrolysis-driven reactions is proportional to the [ATP]/[ADP] concentration ratio. (*c*) The dependence of the relative value of positional error *σ* on the [ATP]/[ADP] concentration ratio for different total protein copy number *N*. Each data point is obtained by averaging five Gillespie simulations. At a given [ATP]/[ADP] ratio, the Turing pattern disappears when *N* ≥ *N*_max_. The inset shows that the maximum protein copy number *N*_max_ (relative to that at the reference [ATP]/[ADP] ratio) increases with the [ATP]/[ADP] ratio. Details of the model and the parameters used are given in the electronic supplementary material.

The accurate spatial clustering of DNA-attached MukBEF complex is critical for chromosome organization. Based on functional and structural studies, a simple ‘rock-climber’ model was proposed by Badrinarayanan *et al.* [21] to explain the working mechanism of the MukBEF complex. Here, we use this model to study the role of energy dissipation in the pattern formation. As illustrated in figure 5*a*, without binding to ATP, a MukB dimer remains at its ‘open’ conformation, which cannot attach to DNA. Once an open MukB dimer binds with ATP, the MukBEF dimer transforms to a ‘close’ conformation, which can dimerize with another closed MukBEF dimer to form a ‘dimer of dimer’ (DD). A DD can attach to DNA by hydrolysing ATP in one of its dimers, which leads to a conformational change from the close state to the open state in that dimer allowing it to capture DNA. This DNA-capturing process is highly cooperative [47], i.e. it is enhanced by having other MukB DDs nearby. Once the MukB dimer captures DNA, the attachment to DNA becomes tighter when it binds to ATP and returns to the close conformation. Once bound to DNA, the MukBEF DD becomes relatively immobile, i.e. the diffusion of the DNA-bound MukB DD is much slower. However, a DNA-bound MukB dimer can detach from DNA by hydrolysing its bound ATP, which changes the dimer to its open conformation and releases DNA, and the next cycle of capturing-and-releasing can start again. In the case when both ATP molecules bound to the DD are hydrolysed (almost) simultaneously, besides releasing the attached DNA, the DD can also de-dimerize to form two separate open dimers. These open dimers have to bind with ATP and dimerize to become functional again.

As shown in figure 5*b*, the reaction network in the Muk system has five chemical species (nodes), but it contains two reaction cycles just like the three-species model shown in figure 1*b*. In the five-species model, *X*_2_ and *X*_1_ represent the closed DD that are bound to the DNA or not, respectively; and *X*_3_ represents the open MukBEF dimer. To describe the effects of ATP hydrolysis, we introduce two intermediate species *M*_1_ and *M*_2_ right after each ATP hydrolysis reaction in the DD loop (*X*_1_ → *M*_1_ → *X*_2_ → *M*_2_ → *X*_1_). The red lines in figure 5*b* represent all the reactions that are driven by ATP hydrolysis. For these ATP hydrolysis-driven reactions, the ratio of the forward reaction rate and the backward reaction rate is proportional to the ATP/ADP ratio, e.g. $k_{1}^{+}/k_{1}^{-}\propto [\mathrm{ATP}]/[\mathrm{ADP}]$. The DNA-free species *X*_1_, *X*_3_ and *M*_2_ are assumed to have the same faster diffusion constant, whereas the DNA-bound species *M*_1_ and *X*_2_ are assumed to have the same slower diffusion constant. See electronic supplementary material for details of the model.

We studied behaviours of this model of the Muk system for different [ATP]/[ADP] ratio, which serves as a proxy for energy dissipation in the system. As shown in figure 5*c*, Turing pattern emerges in a wide range of [ATP]/[ADP] ratio and the positional error *σ* of Turing pattern decreases when the [ATP]/[ADP] ratio increases. Furthermore, for a higher [ATP]/[ADP] ratio, the pattern is more robust to variations in total MukB protein copy number. In particular, the largest MukB protein copy number *N*_max_, below which Turing pattern is stable, increases with the [ATP]/[ADP] ratio (see inset of figure 5*c*). When the [ATP]/[ADP] ratio is smaller than its lower bound (the leftmost points in figure 5*c*), the pattern can still form but with a different stripe number, which is also biologically non-functional. Although the quantitative values for *σ* in our model depend on the parameters we used in the simulations, which may not be the same as in the real experiments, the general trend we predicted, that positional error *σ* decreases with [ATP]/[ADP] ratio and *N*_max_ increases with [ATP]/[ADP] ratio, should still hold. These general predictions on the dependence of precision and robustness of Turing pattern on energy dissipation may be tested in future experiments by varying the ATP/ADP ratio in the system.

## 4. Conclusion and discussion

Accurate spatial organization is critical for many biological processes and functions. However, spatial patterns can fluctuate and even become unstable due to strong noise in small biological systems. In this paper, we investigated whether and how energy dissipation in the underlying non-equilibrium reaction–diffusion systems is related to accuracy and robustness of the spatial pattern by studying a three-species reaction–diffusion model first proposed by Murray & Sourjik [11] motivated by realistic biological systems. The Murray–Sourjik model can be considered as the minimal model capable of generating self-positioned Turing pattern in small biological systems. In addition to the two chemical species in standard Turing pattern models, the additional chemical species (*X*_3_) is needed to create an effective non-local (inter-stripe) interaction over a long timescale to stabilize the specific (targeted) Turing pattern. However, given that the stabilizer specie *X*_3_ only acts at long timescale, the additional energy cost (related to the $1/\Gamma^{\prime }$ cycle in figure 1*b*) for enabling the long timescale stabilization is much smaller than the energy cost of generating and maintaining the Turing pattern (related to the 1/Γ cycle in figure 1*b*). Therefore, the inclusion of such additional chemical species for long timescale stabilization of the Turing patterns represents an efficient design for generating self-positioned Turing patterns, which may be a general feature in realistic small reaction–diffusion systems responsible for accurate spatial ordering such as the Muk system.

We showed that there is a critical (minimum) energy cost (*W*_*c*_) to create and maintain a Turing pattern and *W*_*c*_ decreases as the ratio of the diffusion constants (*d*) increases and it saturates to a finite value as *d* → ∞. As the energy cost increases beyond *W*_*c*_, the spatial accuracy of the Turing pattern increases. A general trade-off relation (equation 3.8) between spatial error *σ* and the energy cost is obtained by analysing phase dynamics of the spatial pattern. In a finite system, we found that the positional error has a distinctive U-shape dependence on total molecule number *N*, and the Turing pattern is stable only in a finite range of *N*. A higher dissipation leads to a wider range of *N* over which the spatial pattern is stable and thus enhances the robustness of the Turing pattern against biologically realistic molecule number variations. We have used this theoretical framework to study the Muk system responsible for DNA segregation in *E. coli* by using a realistic five-species model. Consistent with the theoretical results from the simple three-species model, we found that the Turing pattern becomes more accurate, and it exists in a wider range of *N* as the ATP/ADP ratio increases, both of which can be tested in future experiments. We believe the relationship between energy cost and the accuracy and robustness of the self-positioned Turing pattern is general for small reaction–diffusion systems.

In Turing patterns, spatial regularity arises in a homogeneous system based on an elegant reaction–diffusion (RD) mechanism that depends on the interplay between nonlinear activator–inhibitor chemical reactions and the different diffusion constants for the activator and inhibitor species in the system. There is, however, another class of more direct mechanisms for pattern formation based on pre-existing asymmetry in the system, e.g. a sustained chemical gradient(s) across the entire length of the system. The representative model is the positional information (PI) model (also known as the french flag model) first proposed by Wolpert [48], which has been verified in developmental pattern formation in *Drosophila* [49] and other organisms. These two mechanisms of pattern formation are obviously quite different and they apply to different biological systems (see [50] for a recent review).

These two mechanisms (RD an PI) are also different in terms of their energy cost. In the RD mechanism, the total number of molecules is conserved, and we showed that most of the energy is spent on driving the chemical reaction cycles that convert the molecules from one form to another, which gives rise to the pattern formation. The fraction of energy cost used to overcome diffusion for maintaining the spatial gradient is small. On the other hand, for the PI mechanism, the morphogene molecules have a finite lifetime, and most of the energy is used for synthesizing the morphogen molecules for maintaining the morphogene gradient. In particular, the localized synthesis of the morphogene protein molecule and its global degradation lead to a sustained morphogene gradient, which provides the positional information that can be read off by a downstream mechanism for pattern formation. However, despite the differences between the two mechanisms, as recently reported by Song & Hyeon [51], there is an accuracy–cost trade-off relation in the PI mechanism, which is similar to what we found for the RD mechanism. This raises the question whether there is an universal relation between energy cost and accuracy in pattern formation systems independent of details of the underlying mechanisms, which may provide an interesting direction for future study. In general, we believe the theoretical framework based on non-equilibrium thermodynamics provides a novel lens for investigating biological systems in search of possible unifying principles.

## Data Availability

The codes are available from the GitHub repository: https://github.com/Phyzch/Turing_pattern. The data are provided in electronic supplementary material [52].

## Endnote

1 Subscript ‘0’ is used to represent critical value in an infinite system and subscript ‘*c*’ is used to represent critical value in a finite system.
